# Regulatory B cells and *SIVA1-LAG3* immune suppression predict poor prognosis in prostate cancer

**DOI:** 10.17305/bb.2026.14056

**Published:** 2026-04-10

**Authors:** Weicheng Tian, Yize Li, Jinchuang Li, Chenyu Liang, Shanghua Cai, Biyan Wen, Zeheng Tan, Zhenjie Wu, Yulin Deng, Zige Liu, Yongding Wu, Haiyin Xiao, Weide Zhong, Huichan He, Chitin Hon, Jianheng Ye

**Affiliations:** 1 Department of Urology, Guangzhou First People’s Hospital, Guangzhou Medical University, Guangzhou, Guangdong, China; 2 State Key Laboratory of Quality Research in Chinese Medicine, Macau University of Science and Technology, Taipa, Macau, China; 3 Guangzhou Laboratory, Guangzhou International Bio Island, Guangzhou, Guangdong, China; 4 Guangdong Provincial Key Laboratory of Urology, the First Affiliated Hospital of Guangzhou Medical University, Guangzhou Medical University, Guangzhou, Guangdong, China; 5 Guangdong Key Laboratory of Clinical Molecular Medicine and Diagnostics, Guangzhou First People’s Hospital, School of Medicine, South China University of Technology, Guangzhou, Guangdong, China; 6 Department of Urology, The First Dongguan Affiliated Hospital, Guangdong Medical University, Dongguan, Guangdong, China; 7 Respiratory Disease AI Laboratory in Epidemic Intelligence and Applications of Medical Big Data Instruments, Faculty of Innovation Engineering, Macau University of Science and Technology, Taipa, Macau SAR, China

**Keywords:** Prostate cancer, regulatory B cells, Breg-related prognostic genes, SIVA1, lymphocyte-activation gene 3, tumor microenvironment, immunosuppression

## Abstract

Prostate cancer (PCa) is among the most prevalent malignancies affecting men globally. The immunosuppressive characteristics of the tumor microenvironment significantly hinder the effectiveness of immunotherapeutic strategies. B cells have a dual role in immune responses, with regulatory B cells (Bregs) promoting immune tolerance through the secretion of immunosuppressive cytokines, facilitating tumor immune evasion. However, the specific role of Bregs in PCa remains inadequately understood. In this study, we systematically identified Breg subpopulations in PCa using single-cell RNA sequencing (scRNA-seq), multi-omics analysis, and machine learning techniques, while also investigating their association with clinical prognosis. Our analysis revealed that increased infiltration of Bregs is strongly correlated with shorter biochemical recurrence-free survival (BCRFS). Additionally, higher expression levels of *SIVA1* apoptosis-inducing factor (*SIVA1*) were positively associated with the immune checkpoint lymphocyte-activation gene 3 (*LAG3*). Utilizing Breg-related prognostic genes (BRPGs), we developed a novel model for predicting BCRFS, which we validated across multiple independent cohorts. Further immunohistochemical analysis confirmed the positive correlation between elevated SIVA1 expression and poor prognosis, as well as its association with *LAG3*. Collectively, these findings provide new insights into the immune microenvironment of PCa and suggest that Breg-related characteristics and the *SIVA1–LAG3* relationship in tumor cells may represent promising therapeutic targets for future research.

## Introduction

According to recent epidemiological data, prostate cancer (PCa) is the most prevalent malignancy affecting male reproductive and urinary health globally, with incidence and mortality rates ranked second and fifth, respectively [1]. Similar to many other solid tumors, the tumor microenvironment (TME) of PCa is often highly immunosuppressive. This immunosuppressive state is characterized by the presence of suppressive immune cells, including T cells, tumor-associated macrophages (TAMs), myeloid-derived suppressor cells (MDSCs), and fibroblasts secreting factors such as transforming growth factor beta (TGF-β) and adenosine, which together create a “cold tumor” environment that limits the efficacy of immunotherapy [2–4].

The development of PCa is influenced by a complex interplay of genetic, hormonal, and environmental factors [1]. Age is the most significant risk factor, with a marked increase in incidence observed among older men [1]. Genetic predispositions, including mutations in DNA repair genes such as breast cancer susceptibility gene 1/2 (BRCA1/2), also contribute to disease susceptibility [5, 6]. Furthermore, androgen receptor (AR) signaling is critical for tumor initiation and progression, as it promotes cell proliferation and survival [7]. Environmental and metabolic factors may further modulate disease development [8]. These elements not only drive tumor progression but also contribute to the formation of an immunosuppressive TME, thereby influencing the functional state of infiltrating immune cells [9, 10].

B cells, which are pivotal in humoral immunity, can either inhibit or promote tumor progression, contingent on the specific TME [11–13]. They can directly suppress tumor growth by secreting immunoglobulins that neutralize tumor cells and enhance T cell responses, thereby facilitating immune attacks on tumor cells [14]. For instance, in renal cell carcinoma and ovarian cancer, terminally differentiated B cell plasma cells produce anti-tumor antibodies that may mark cancer cells for elimination [15, 16]. Additionally, tumor-infiltrating B cells (TIBs) can form tertiary lymphoid structures (TLSs), which promote T cell infiltration and tumor cell lysis, subsequently enhancing anti-tumor immune responses. The presence of TLSs is correlated with improved prognoses in cancers such as lung and ovarian cancer [17, 18].

Conversely, within the B cell population exists a heterogeneous group known as regulatory B cells (Bregs), which produce immunosuppressive cytokines, including interleukin (IL)-10, IL-35, and TGF-β. Bregs are essential for maintaining immune tolerance and regulating inflammation [19, 20]. By secreting these cytokines, Bregs inhibit effector T cell activation and promote immune tolerance through direct interactions with other immune cells, such as regulatory T cells (Tregs), dendritic cells, macrophages, and natural killer (NK) cells, thereby facilitating tumor immune evasion [21–25]. For example, in gastric cancer, an increase in IL-10-producing Bregs is associated with immunosuppression and poor prognosis [26]. In certain gynecological cancers, including breast, cervical, and ovarian cancers, Granzyme B-secreting regulatory B cells (GraB cells) are found adjacent to T cells, potentially aiding tumor escape from immune attack by fostering immune tolerance [27].

In the context of PCa, although significant research has focused on T cells and immune checkpoints [28–32], the role of B cells as critical humoral immune components remains poorly understood. Previous studies have documented substantial B cell infiltration in PCa tissues, observing a significantly higher number of B cells in malignant tissues compared to benign prostate tissues [33–35]. Additionally, proliferating and clonally expanded B cell subpopulations have been identified in the peripheral blood and lymph nodes of PCa patients, indicating an active anti-tumor immune response [36]. However, considering that B cells can exert both anti-tumor effects (such as antibody production and antigen presentation) [37] and possess potentially tumor-promoting immunosuppressive functions [38], we hypothesize that the immunosuppressive Breg subpopulation may play a more prominent role in immune suppression within the TME.

Research on Bregs in PCa is still limited, with systematic phenotypic and functional analyses lacking. This study aims to systematically identify the Breg subpopulation in PCa using single-cell transcriptomics data and multi-omics validation, while exploring its role in immune suppression within the TME. Based on Breg-related prognostic genes, we constructed a Breg-related prognostic model (BRPGs). Furthermore, we identified the key gene SIVA1, which is closely associated with Bregs, revealing that high SIVA1 expression correlates significantly with shorter biochemical recurrence-free survival (BCRFS) in PCa. Subsequent immunohistochemistry (IHC) and Western blot experiments confirmed a positive correlation between SIVA1 expression and the immune checkpoint molecule Lymphocyte-Activation Gene 3 (LAG3). Inhibition of SIVA1 downregulated LAG3 expression in PCa cell lines, suggesting a potential association between SIVA1 and LAG3 in tumor cells. The direct contribution of this relationship to immune suppression within immune cell compartments warrants further investigation.

## Materials and methods

### Data collection

The single-cell RNA sequencing (scRNA-seq) data utilized in this study were obtained from GSE181294. According to the original publication, this cohort comprised 19 treatment-naive patients diagnosed with prostate adenocarcinoma who underwent radical prostatectomy [39]. Detailed patient sample information is provided in Table S1. Bulk transcriptomic data from seven independent PCa cohorts—The Cancer Genome Atlas Prostate Adenocarcinoma (TCGA-PRAD), GSE21034, GSE54460, GSE107299, E-MTAB-6128, DKFZ, GSE70769, and GSE94767—were obtained from the PCaDB resource. This database provides uniformly processed PCa transcriptomic datasets curated from public repositories, including TCGA (http://portal.gdc.cancer.gov/), Gene Expression Omnibus (GEO, https://www.ncbi.nlm.nih.gov/geo/), ArrayExpress (https://www.ebi.ac.uk/arrayexpress/), and cBioPortal (https://www.cbioportal.org/ [40–46]). These cohorts included bulk transcriptomic profiles generated through either RNA sequencing or microarray platforms, along with corresponding clinical follow-up data, which were utilized for model construction and external validation. The Breg-related gene set was compiled from previous literature [47, 48] (Table S2).

### Bulk transcriptomic data preprocessing and harmonization

To enhance comparability across cohorts generated from different platforms, we used the uniformly processed gene expression matrices provided by PCaDB. According to the PCaDB processing pipeline, RNA sequencing (RNA-seq) datasets were normalized using the trimmed mean of M-values (TMM) method implemented in the edgeR package, while microarray datasets were normalized using the robust multi-array average (RMA) method. Gene annotations were harmonized to Ensembl gene IDs across datasets. For genes represented by multiple probes in microarray datasets, the probe with the largest interquartile range (IQR) was selected as the representative probe. The processed expression matrices were provided on a comparable log2 scale and were directly used for downstream analyses in this study. No additional cross-cohort batch correction or re-normalization was performed, as each external cohort was evaluated independently rather than merged into a single validation set.

### Data processing

The “Seurat” package in R was employed to process the scRNA-seq data. The raw expression matrix was first converted into a Seurat object, followed by rigorous quality control (QC) procedures. The QC criteria were as follows: retention of cells with gene expression counts (nFeature_RNA) between 300 and 7000, mitochondrial gene percentage (mt_percent) below 40%, hemoglobin gene percentage (HB_percent) below 3%, and sequencing depth (nCount_RNA) greater than 600 but lower than the 97th percentile of cell counts. Cells expressing fewer than 1000 genes were excluded, and only genes detected in at least 10 cells were retained for subsequent analysis. The “FindVariableFeatures” function identified the top 2000 most variable genes, which were subsequently used for principal component analysis (PCA). The “harmony” algorithm was utilized to integrate multiple datasets and mitigate batch effects. After batch correction, Uniform Manifold Approximation and Projection (UMAP) was employed for dimensionality reduction and visualization. Cell clustering was performed using the K-nearest neighbor (KNN) method with a resolution of 0.8. Cell subpopulations were annotated and identified based on previously reported cell-type-specific marker genes [39, 49–51] (Table S3). Pathway enrichment analysis was conducted using the “addmodulescore” function in Seurat.

### CellChat

We utilized the CellChat package to analyze intercellular communication between B cell and T cell subpopulations in the PCa scRNA-seq data. Initially, we extracted the expression matrix and cell classification information from the Harmony-integrated Seurat object to create a CellChat object. Using the paracrine/autocrine signaling pathway database from CellChatDB as a reference, we calculated the probability of ligand-receptor interactions between cells using the “computeCommunProb” function. To ensure the robustness of the analysis, we retained only genes expressed in at least 10 cells and employed statistical methods to determine the significance of intercellular communication. Visual analyses were conducted to explore differences in intercellular communication between B cell and T cell subpopulations with high vs low Breg scores.

### BayesPrism analysis

BayesPrism was utilized to investigate the correlation between Bregs subpopulations and clinical data. The software was installed from the GitHub repository (https://github.com/Danko-Lab/BayesPrism) [52]. Single-cell-derived Bregs signatures identified from scRNA-seq data served as reference expression profiles for the deconvolution of bulk RNA-seq datasets using BayesPrism. The software computes an overall score for each cell type by aggregating deconvolution scores across various cell states within that type. After calculating scores for distinct B-cell subpopulations from each patient’s bulk transcriptomic data, we utilized the Bregs subpopulation (cluster 7) score to conduct survival analyses and assess its associations with clinical characteristics, thereby evaluating its potential impact on patient prognosis.

### Clinical endpoint definition

In this study, BCRFS was defined as the primary endpoint across all cohorts. BCRFS was defined as the time from initial treatment (e.g., radical prostatectomy) or the specified time origin in each dataset to the first occurrence of biochemical recurrence. Biochemical recurrence (BCR) was defined as a prostate-specific antigen (PSA) level ≥ 0.2 ng/mL confirmed by two consecutive measurements, when such information was available. For publicly available datasets lacking detailed PSA-based definitions, the original “time to BCR” or equivalent endpoint annotations provided by the dataset were utilized as proxies for BCRFS. Patients without documented recurrence events were treated as censored at the time of last follow-up. The time origin, event definition, and censoring strategy were aligned with the clinical annotations of each individual dataset.

### Kaplan–Meier survival analysis

The Kaplan–Meier method was employed to estimate BCRFS. Patients were stratified into four groups based on high or low Bregs scores and SIVA1 expression levels, generating survival curves that indicated censored observations with tick marks. Differences among groups were assessed using the log-rank test. To categorize patients into high- and low-expression groups based on the Bregs score, the optimal cut-off value was determined using the surv_cutpoint function from the survminer R package (version 0.4.6). This method, which relies on maximally selected rank statistics [53], identifies the threshold that most effectively differentiates patients in Kaplan–Meier survival analysis.

### High-dimensional weighted gene co-expression network analysis (hdWGCNA)

During the module identification process, we computed the gene expression correlation matrix and constructed a weighted co-expression network, identifying multiple functional gene modules. Module-trait relationship analysis was conducted to select key modules associated with the Bregs subpopulation. We then identified hub genes based on intramodular connectivity. In the pink module (M1) of the Bregs subpopulation, 74 potential hub genes were identified, which may be related to PCa metastasis. The hdWGCNA analysis adhered to the guidelines published in Nature Genetics (2021) [54] to ensure scientific rigor and reproducibility.

### Machine learning combinations

To identify key genes related to Bregs with potential prognostic value, we used the TCGA-PRAD cohort as the training set and included several independent external validation datasets (GSE21034, GSE54460, GSE107299, E-MTAB-6128, DKFZ, GSE70769, and GSE94767) to enhance the robustness and applicability of the findings (Table S4). We applied ten popular machine learning algorithms, including Lasso regression, Ridge regression, CoxBoost, Elastic Net, Stepwise Cox regression, Random Survival Forest (RSF), Partial Least Squares Cox regression (plsRcox), SuperPC, Generalized Boosted Regression Models (GBM), and Survival Support Vector Machine (Survival-SVM), for feature selection and modeling. Using a 10-fold cross-validation framework, a total of 117 algorithm combinations were evaluated in the TCGA-PRAD training cohort to identify the optimal model. Model performance was assessed using the concordance index (C-index) across the training set, internal validation set, and multiple independent external validation cohorts. Based on the best-performing combination strategy, a BRPGs risk model was established. Univariate Cox regression analysis evaluated the prognostic significance of candidate genes, and multivariate Cox regression was conducted for selected genes to assess their independent effects. The study primarily relies on machine learning–based modeling approaches, which account for multivariate relationships among variables. The risk score was calculated as a linear combination of gene expression values weighted by the regression coefficients derived from the final selected training model, where βi represents the coefficient of gene i, and Expressioni represents its expression level. The final BRPGs score was defined as: BRPGs score ═ (−0.2734 × *GOPC*) + (0.5275 × *OSBPL8*) + (0.2156 × *DNAJC2*) + (0.2333 × *ITGA4*) + (0.2687 × *ARRB2*) + (−0.3266 × *MBNL1*) + (0.2270 × *SON*) + (0.2598 × *ZMAT1*) + (−0.1861 × *KIF5B*) + (0.4694 × *SIVA1*). Gene expression matrices were obtained directly from the uniformly processed PCaDB resource and were used for risk score calculation without additional cross-cohort batch correction, centering, or scaling. For all external validation datasets, the regression coefficients derived from the training cohort were fixed and directly applied without re-fitting or recalibration. Specifically, the BRPG risk score for each sample was calculated as the sum of the normalized expression value of each gene multiplied by its corresponding coefficient from the final training model. This fixed-coefficient strategy ensured that the external validation represented a true independent validation process and reduced the risk of overfitting while maintaining comparability across cohorts.

### Nomogram construction

To improve the clinical relevance of the model, we integrated clinical pathological features—such as PSA levels, Gleason scores, and American Joint Committee on Cancer (AJCC) stage—with risk scores to develop a comprehensive nomogram for predicting BCRFS in patients. BCR, the event underlying BCRFS, was defined as a PSA level ≥ 0.2 ng/mL confirmed by two consecutive measurements [55]. The calibration curve was employed to validate the concordance between model-predicted outcomes and actual clinical observations. Furthermore, Receiver Operating Characteristic (ROC) curves were utilized to assess the nomogram’s accuracy in predicting BCRFS. Additionally, clinically established models (CEMs) were developed using common clinical indicators (such as age, tumor stage, PSA, Gleason score, etc.) with the R package ‘survival’. The pec R package was used to calculate the Brier scores of the nomogram at one-year, three-year, and five-year follow-ups, further validating the model’s accuracy [56].

### Enrichment analysis

To gain insights into the biological functions and signaling pathways associated with Bregs-related genes, we conducted Gene Ontology (GO) and WikiPathways pathway enrichment analyses using the Metascape online tool. Metascape (http://metascape.org) is a comprehensive platform for gene function annotation and analysis, integrating multiple authoritative databases to provide systematic gene functional analysis. We set the statistical significance threshold at *P* < 0.01, with a minimum gene enrichment count of ≥3 and an enrichment factor (observed gene count to expected gene count ratio) >1.5. We then clustered and integrated terms based on gene similarity, selecting the most statistically significant terms from each cluster as representatives to refine and clearly present the main functional modules and pathways involved in Bregs-related genes.

### IHC

IHC was performed on tissue microarrays (TMAs) consisting of 60 PCa samples and paired adjacent normal tissues (Catalog No.: HProA120Su01, Shanghai Xinchao Biotechnology Co., Ltd.). Detailed TMA information is available at https://www.superchip.com.cn/biology/tissue.html. To enhance accessibility, detailed clinicopathological and follow-up information of the TMA cohort is provided in Table S5. IHC staining was conducted according to the methods described in our previous study [57]. Specific antibodies (1:3000 dilution, Catalog No. A6326, ABCLOINAL, Rabbit polyclonal antibody) were employed to detect SIVA1 protein expression in PCa tissues. Based on the average IHC score of 4.6 in PCa tissues, the TMA cohort patients were classified into high and low SIVA1 expression groups.

### HuPSA-MoPSA platform validation

To further validate the expression pattern of SIVA1 in PCa, we performed cross-validation using the HuPSA-MoPSA platform (https://pcatools.shinyapps.io/HuPSA-MoPSA/) [58]. This platform provides transcriptomic data from various PCa cell lines.

### Cell culture and small interfering RNA (siRNA) transfection

Human PCa cell lines C4-2 and C4-2B were obtained from a commercial source and authenticated by short tandem repeat (STR) profiling prior to use. All cell lines were routinely tested and confirmed to be mycoplasma-free. Cells were cultured in RPMI-1640 medium (MA0215, Meilunbio, Dalian, China) supplemented with 10% fetal bovine serum (HF1032-05, Holocene, Guangzhou, China) and 1% penicillin/streptomycin (15140-122, Gibco, Grand Island, NY, USA). Cells were maintained at 37^∘^C in a humidified incubator with 5% CO2. SIVA1-specific siRNAs and negative control siRNA were procured from GenePharma Co., Ltd. (Shanghai, China). C4-2 and C4-2B cells were transfected at 70%–80% confluence using siRNA-mate plus transfection reagent (GenePharma, cat. no. G04026) per the manufacturer’s instructions, with a final siRNA concentration of 75 nM. The siRNA sequences are listed in Table S6. All experiments were conducted with at least three independent biological replicates.

### Western blot analysis

Total protein was extracted from C4-2 and C4-2B cells following washing with pre-cooled phosphate-buffered saline (PBS). Equal amounts of protein were separated by sodium dodecyl sulfate–polyacrylamide gel electrophoresis (SDS-PAGE) and subsequently transferred onto membranes. After blocking, the membranes were incubated overnight at 4^∘^C with the following primary antibodies: glyceraldehyde-3-phosphate dehydrogenase (GAPDH) (1:10,000; ab8227, Abcam, Cambridge, UK), SIVA1 (1:1,000; 67565-1-Ig, Proteintech, Chicago, IL, USA), and LAG3 (1:1,000; 21646-1-AP, Proteintech, Chicago, IL, USA). The membranes were washed with tris-buffered saline with Tween 20 (TBST) and incubated with horseradish peroxidase (HRP)-conjugated secondary antibodies at room temperature for 1 hour. Protein bands were visualized using an enhanced chemiluminescence detection system. Band intensities were quantified using ImageJ software developed by the National Institutes of Health (NIH) in Bethesda, MD, USA. The gray value of each target band was normalized to that of the corresponding GAPDH band, with the normalized value of the control group set to 1.0. All experiments were performed with at least three independent biological replicates.

### Statistical analysis

All statistical analyses and data visualizations in this study were conducted using R software (version 4.3.2), GraphPad Prism (version 9.5.1), and the comprehensive online analysis platform CNSknowall (https://cnsknowall.com). Differences between groups for continuous variables were evaluated using the non-parametric Mann–Whitney *U* test. BCRFS survival analysis was performed using the Kaplan–Meier method to generate BCRFS survival curves, with the Log-rank test applied to assess the significance of group differences. Additionally, forest plots and correlation lollipop plots were generated using the CNSknowall online analysis platform, which integrates data analysis, statistical testing, and advanced visualization functions. A two-tailed *P*-value of less than 0.05 was considered statistically significant for all analyses. Due to the retrospective nature of this study, based on publicly available datasets and pre-existing tissue samples, no formal sample size calculation was performed; sample sizes were determined by data availability in each dataset. To ensure the reliability of the results, multiple independent cohorts were included, and external validation was conducted across datasets.

## Results

### The single-cell transcriptomic landscape of PCa reveals a unique Bregs subpopulation

The workflow of this study is illustrated in Figure 1A. To explore cellular heterogeneity within the PCa immune microenvironment, we systematically analyzed the scRNA-seq dataset GSE181294, which includes data from 19 treatment-naive PCa patients. UMAP dimensionality reduction identified 11 major cell types, including B cells, T cells, fibroblasts, myeloid cells, and endothelial cells (Figure 1B). Cell annotation was based on classical marker genes and visualized through bubble plots, where different cell populations exhibited distinct gene expression profiles (Figure 1C). Among the B-cell population, we identified a subpopulation characterized by high expression of IL10 and other typical Bregs marker genes. The Bregs-associated gene set was defined based on the overlap between B10 cells (B10)-associated genes and genes involved in the negative regulation of the immune system [47, 48] (Figure 1D). Notably, the inclusion of checkpoint-related genes reflects the functional immunosuppressive state of Bregs rather than contamination from other immune cell types. GO enrichment analysis revealed that these genes are primarily involved in immune regulation, cytokine signaling, and inflammatory responses (Figure 1E). Further subpopulation clustering of B cells (Figure 1F and G) and Bregs scoring using the AddModuleScore algorithm revealed the distribution of Bregs across the UMAP plot (Figure S1A). A bubble plot visualized IL10 expression in each B cell subpopulation, with cluster 7 exhibiting the highest *IL10* expression, thus identifying it as the Bregs population (Figure S1C). Immune suppression scoring of B cell subpopulations indicated that greater immunosuppressive activity was enriched in the Bregs subset, suggesting its critical role in shaping the immunosuppressive microenvironment of PCa (Figure 1H and I). Further analysis of immunosuppressive molecules revealed that the Bregs subpopulation demonstrated increased expression levels of classical immunosuppressive cytokines, including IL-10 and transforming growth factor beta 1 (TGF-β1). Additionally, this subpopulation exhibited heightened expression of immune checkpoint molecules such as *LAG3*, T-cell immunoreceptor with Ig and immunoreceptor tyrosine-based inhibitory motif (ITIM) domains (TIGIT), and hepatitis A virus cellular receptor 2 (*HAVCR2*) (Figure S1B). The inclusion of checkpoint-related genes underscores the functional immunosuppressive state of Bregs. These findings suggest that Bregs may contribute to the establishment of an immunosuppressive TME in PCa, potentially through the secretion of inhibitory cytokines and engagement with immune checkpoint pathways in interactions with other immune cells. However, further validation through functional experiments is warranted.

**Figure 1.**
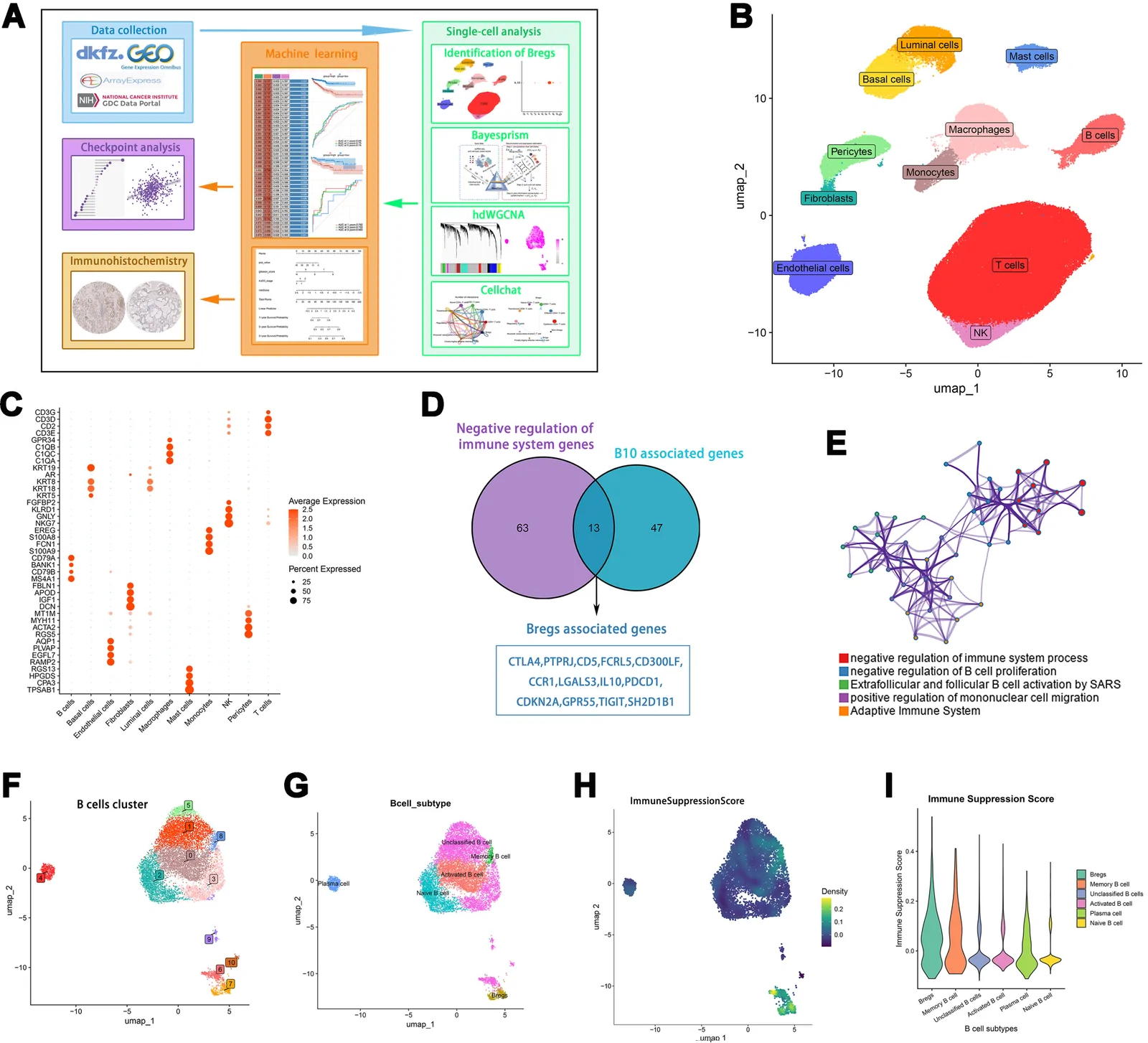
**Single-cell transcriptomic analysis identifies an immunosuppressive Bregs subpopulation in PCa.** (A) Schematic overview of the study workflow, integrating public transcriptomic data collection, single-cell analysis for Bregs discovery, machine-learning-based prognostic modeling, checkpoint analysis, and immunohistochemical validation. (B) UMAP projection of the GSE181294 dataset from 19 treatment-naïve PCa patients, identifying 11 major cellular populations, including T cells, B cells, natural killer cells, macrophages, myocytes, fibroblasts, pericytes, endothelial cells, basal cells, luminal cells, and mast cells. (C) Bubble plot of canonical marker genes used for cell-type annotation; dot size indicates the proportion of cells expressing each marker, and color intensity indicates the average expression level. (D) Venn diagram showing the overlap between genes involved in the negative regulation of the immune system and previously reported regulatory B-cell-associated genes, yielding a 13-gene Bregs-associated signature for downstream analyses. (E) Functional enrichment network of the Bregs-associated gene set, highlighting biological programs predominantly related to immune regulation, inflammatory signaling, B-cell regulatory activity, and cell migration. (F) UMAP of reclustered B cells showing the intralineage heterogeneity of the B-cell compartment. (G) Annotation of B-cell subclusters into Bregs, memory B cells, unclassified B cells, activated B cells, plasma cells, and naïve B cells; the Bregs subset was defined by its immunoregulatory transcriptional profile and high *IL10*-associated features. (H) UMAP feature plot showing the distribution of the immunosuppression score across B-cell subtypes. (I) Violin plot comparing immunosuppression scores among annotated B-cell subtypes, demonstrating enrichment of the suppressive program in Bregs relative to the other B-cell populations. **Abbreviations:** Bregs, regulatory B cells; *IL10*, interleukin 10; UMAP, Uniform Manifold Approximation and Projection.

### The infiltration level of Bregs is significantly associated with poor prognosis in PCa

To investigate the potential clinical significance of Bregs subpopulations in PCa progression, we employed BayesPrism for the deconvolution of bulk RNA-seq data from the TCGA cohort to estimate the proportion of each B cell subpopulation in individual patients. Quality control results demonstrated that this method effectively reduced noise in both scRNA-seq and bulk data (Figure 2A). A heatmap of intercellular correlations revealed the relationships between different subpopulations (Figure 2B). Infiltration analysis of the deconvoluted cells indicated differential infiltration levels of Bregs across patient samples, with significant enrichment observed in certain patients (Figure 2C). Kaplan-Meier analysis of BCRFS indicated that high Bregs infiltration was significantly associated with shorter BCRFS (Figure 2D). Moreover, Bregs enrichment levels were positively correlated with adverse clinical features, such as higher Gleason scores and advanced T/N staging (Figure 2E).

**Figure 2.**
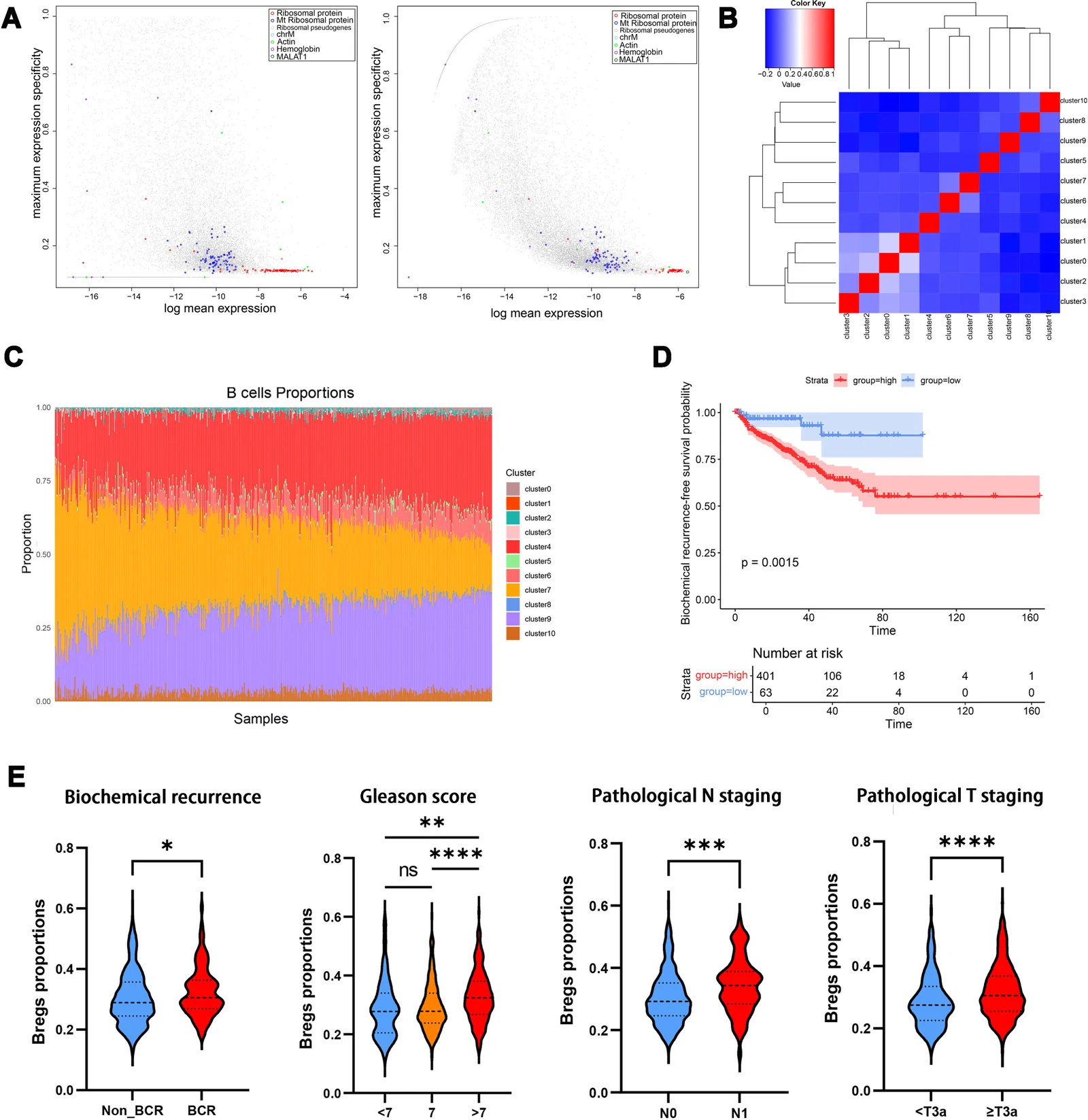
**BayesPrism deconvolution reveals that Bregs infiltration is associated with adverse prognosis and clinicopathological progression in PCa.** (A) BayesPrism quality-control plots for deconvolution of bulk RNA-seq data using the scRNA-seq reference, showing removal of low-quality or noisy genes and improved concordance between bulk and single-cell expression profiles. (B) Hierarchically clustered correlation heatmap of the inferred B-cell subpopulations, illustrating the relationships among deconvoluted clusters. (C) Stacked proportional plot showing the estimated distribution of B-cell subpopulations across individual PCa samples, highlighting interpatient heterogeneity in B-cell composition and variable enrichment of the Bregs-associated cluster. (D) Kaplan–Meier analysis of biochemical recurrence-free survival stratified by the inferred abundance of the Bregs-associated cluster, showing significantly shorter biochemical recurrence-free survival in the Bregs-high group than in the Bregs-low group; shaded areas indicate 95% confidence intervals, and the table below shows the numbers at risk. (E) Violin plots showing the association between inferred Bregs proportions and adverse clinicopathological features, including biochemical recurrence status, Gleason score, pathological N stage, and pathological T stage. Dashed lines indicate the median and interquartile range. Statistical significance is indicated as *P* < 0.05, *P* < 0.01, *P* < 0.001, and *P* < 0.0001; ns, not significant. **Abbreviations:** BCR, biochemical recurrence; BCRFS, biochemical recurrence-free survival; Bregs, regulatory B cells; RNA-seq, RNA sequencing; scRNA-seq, single-cell RNA sequencing.

### hdWGCNA identifies Bregs-specific co-expression modules and key hub genes

To systematically analyze the regulatory network of Bregs subpopulations, we constructed a B cell co-expression network utilizing the hdWGCNA algorithm. Nine co-expression modules were identified using a soft threshold of β ═ 7 (Figure 3A and B). The clustering results for each module and their representative genes are displayed in the figure (Figure 3C). UMAP and bubble plots further revealed significant activation of the M1 (pink) module in Bregs, and regulatory networks for each module were constructed (Figure 3D–F). We extracted potential hub genes from the M1 module and constructed their interaction network (Figure 3G). Additionally, differential analysis between B cell subpopulations and volcano plots visualized differential genes for each B cell subpopulation (Figure 3H). A Venn diagram analysis of differentially expressed genes in Bregs and the M1 module identified 20 functional core genes (Figure 3I).

**Figure 3.**
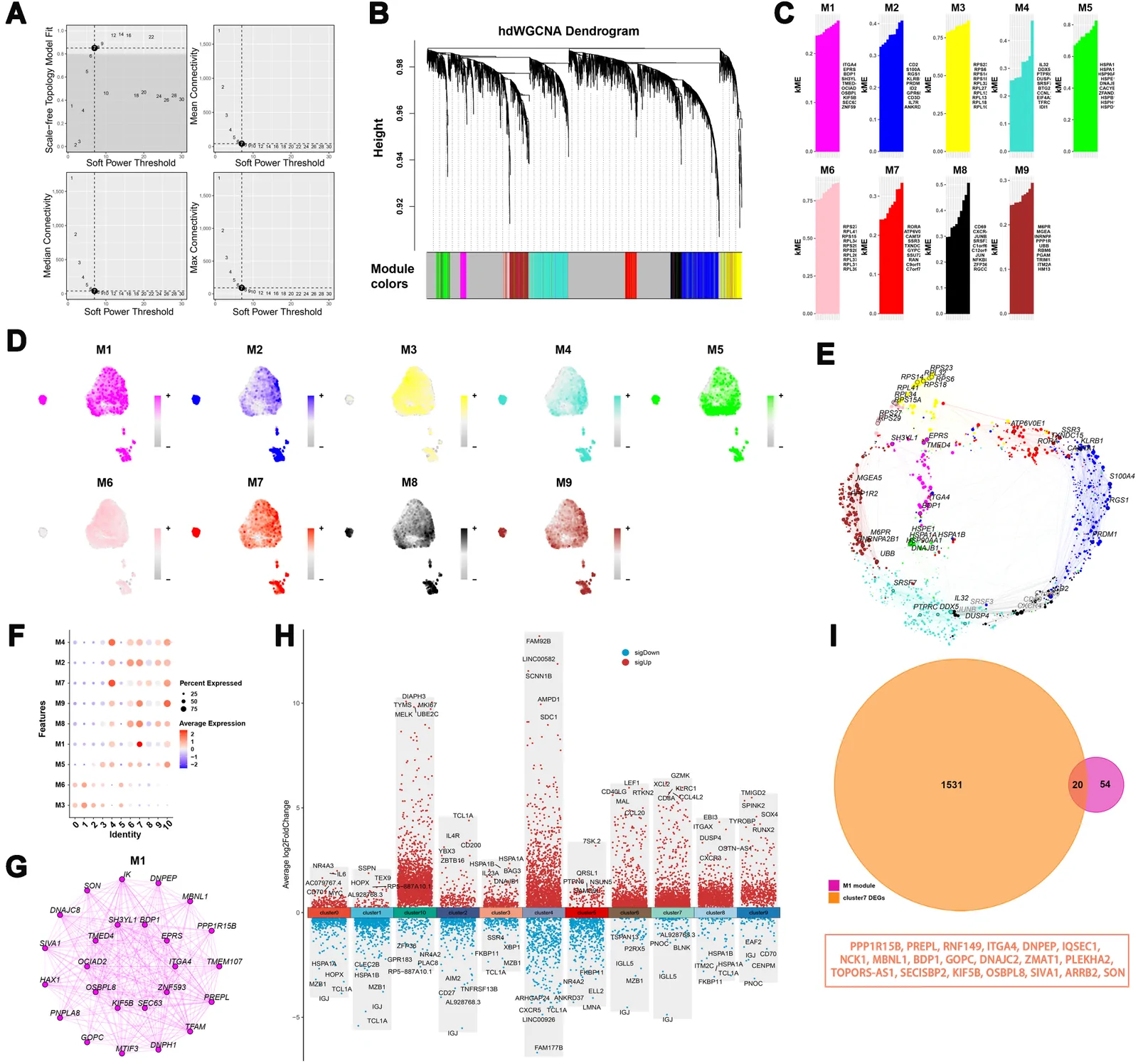
**hdWGCNA identifies Bregs-enriched co-expression modules and core candidate genes in the B-cell compartment of PCa.** (A) Selection of the soft-thresholding power for hdWGCNA based on scale-free topology fit and network connectivity, with β ═ 7 chosen for downstream network construction. (B) Hierarchical clustering dendrogram of B-cell genes showing nine co-expression modules identified by hdWGCNA; the color bar indicates module assignment. (C) Bar plots showing the top 10 genes ranked by intramodular connectivity within each module (M1–M9). (D) UMAP feature plots showing the distribution of module activity across B-cell subpopulations, with prominent enrichment of the M1 (pink) module in the Bregs cluster. (E) Module-specific co-expression network of the B-cell compartment; nodes represent genes colored by module assignment, node size reflects eigengene-based connectivity, and labeled nodes indicate representative hub genes. (F) Dot plot summarizing relative module activity across B-cell subclusters, further highlighting preferential activation of M1 in Bregs. (G) Interaction network of hub genes within the M1 (pink) module. (H) Volcano plots of differentially expressed genes for each B-cell subpopulation compared with the remaining B-cell clusters, with upregulated genes shown in red and downregulated genes shown in blue. (I) Venn diagram showing the overlap between differentially expressed genes in cluster 7 (Bregs) and genes assigned to the M1 module, identifying 20 shared candidate core genes for downstream analyses. **Abbreviations:** Bregs, regulatory B cells; DEGs, differentially expressed genes; hdWGCNA, high-dimensional weighted gene co-expression network analysis; UMAP, Uniform Manifold Approximation and Projection.

### Construction and validation of the BRPGs

To identify clinically relevant Bregs-related biomarkers, we constructed 117 model combinations based on 20 candidate genes and 10 popular machine learning algorithms (Figure S2). The StepCox (both) + ElasticNet (0.9) strategy demonstrated the best C-index performance across validation cohorts (Figure 4A). The BRPGs constructed using this strategy effectively distinguished high- and low-risk patients in the TCGA training cohort, as well as in several external validation cohorts (GSE21034, GSE54460, GSE107299, E-MTAB-6128, DKFZ, GSE70769, and GSE94767). BCRFS Kaplan-Meier survival curves and ROC analysis at 1-, 3-, and 5-year time points exhibited strong predictive performance (Figure 4B–I).

**Figure 4.**
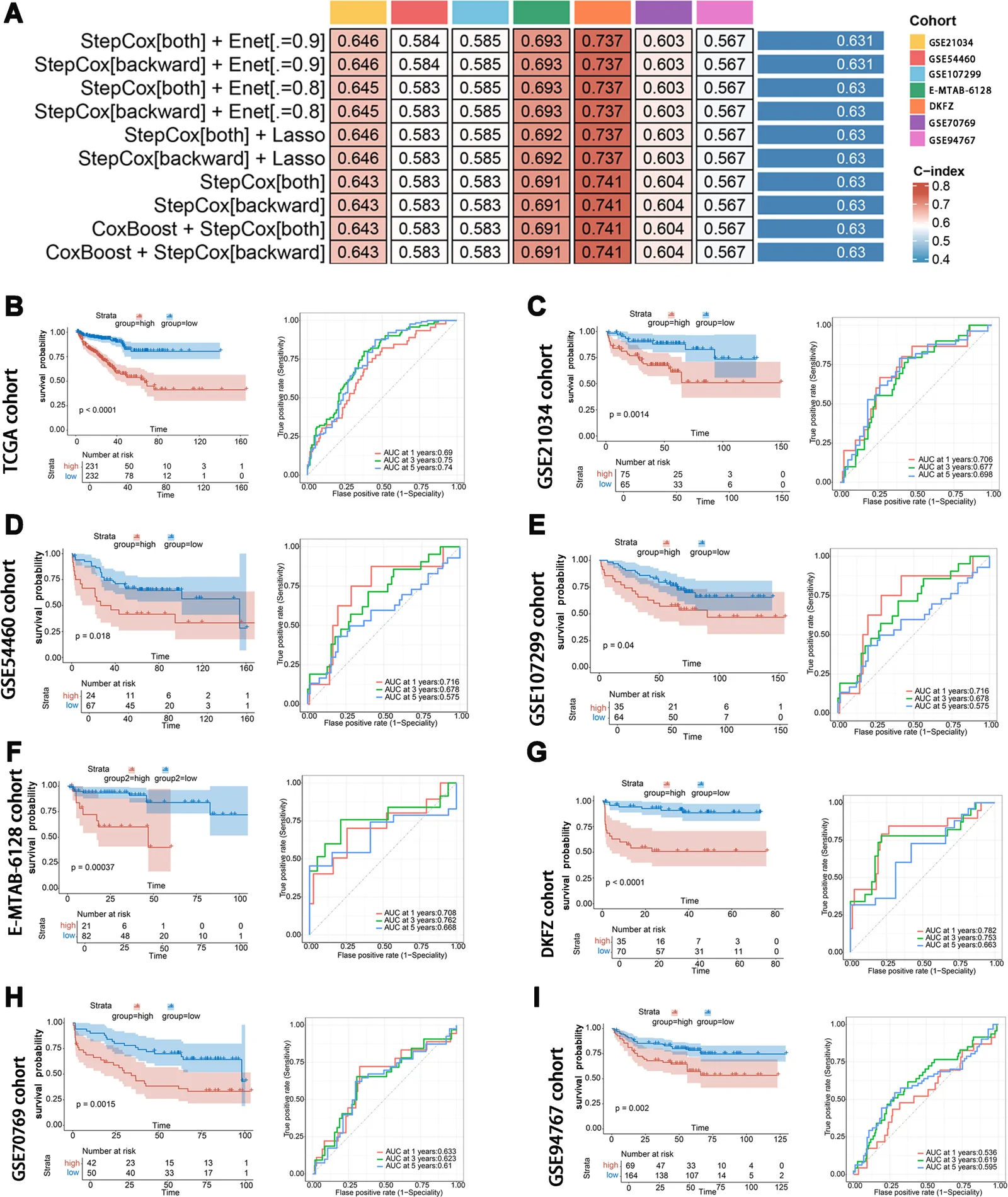
**Machine-learning-based development and multicohort validation of the BRPGs prognostic model for biochemical recurrence-free survival in PCa.** (A) Heatmap showing the top 10 of 117 machine-learning model combinations ranked by concordance index across seven independent validation cohorts. The StepCox (both) + Elastic Net (α ═ 0.9) combination achieved the best overall performance, and the bar on the right summarizes the mean concordance index across cohorts. (B–I) Kaplan–Meier survival curves (left) and time-dependent receiver operating characteristic curves (right) for the BRPGs model in the TCGA training cohort and external validation cohorts: (B) TCGA, (C) GSE21034, (D) GSE54460, (E) GSE107299, (F) E-MTAB-6128, (G) DKFZ, (H) GSE70769, and (I) GSE94767. Stratification by BRPGs score consistently separated patients into high- and low-risk groups for biochemical recurrence-free survival, with poorer outcomes in the high-risk group. Shaded areas represent 95% confidence intervals, and the tables below the survival curves indicate the numbers at risk. Time-dependent receiver operating characteristic analysis further demonstrates the predictive performance of the model at 1, 3, and 5 years across the training and validation cohorts. **Abbreviations:** AUC, area under the curve; BCRFS, biochemical recurrence-free survival; BRPGs, Bregs-related prognostic genes; DKFZ, German Cancer Research Center; ROC, receiver operating characteristic; TCGA, The Cancer Genome Atlas.

**Figure 5.**
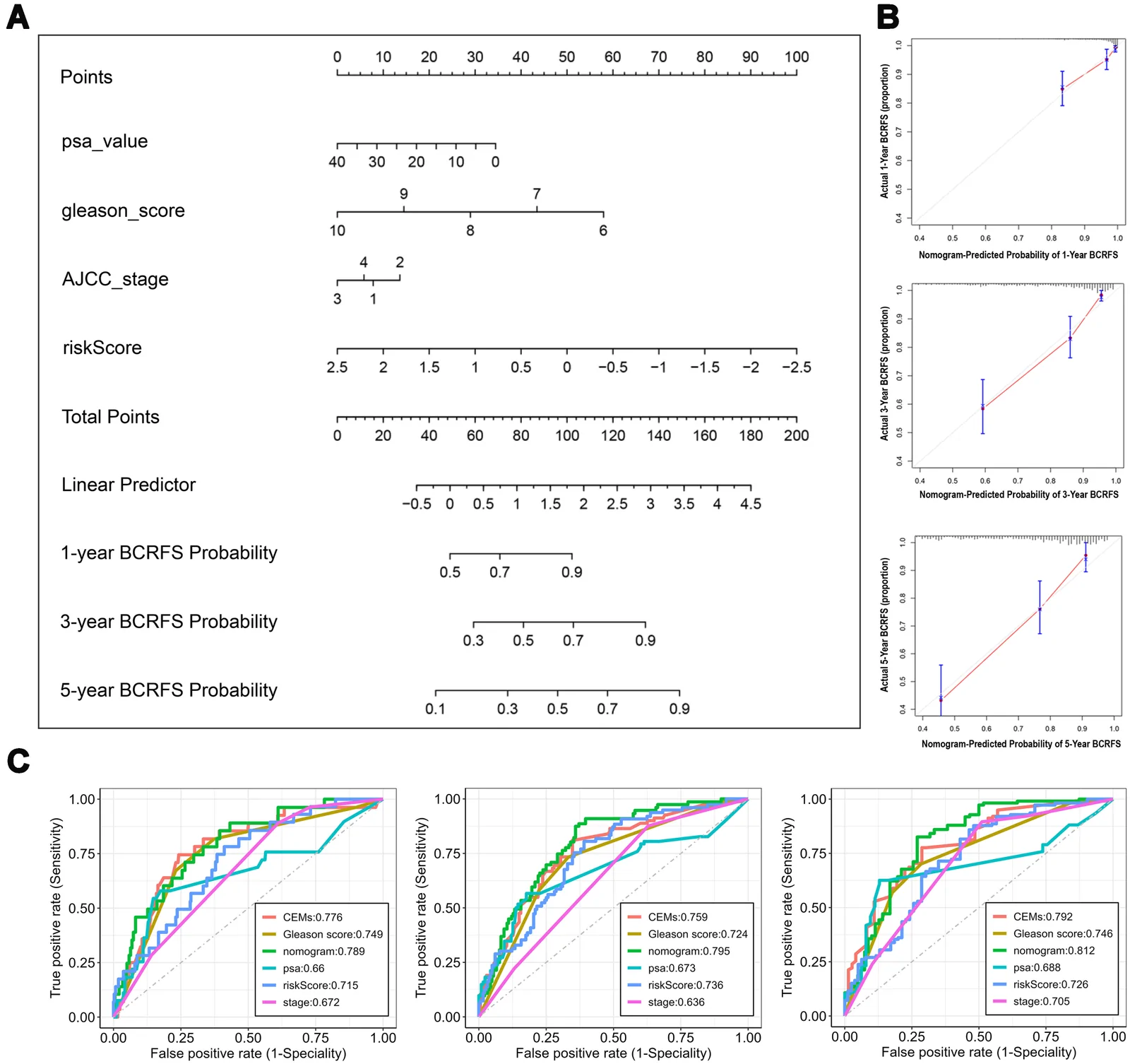
**Nomogram integrating the BRPGs risk score and clinicopathological variables improves prediction of biochemical recurrence-free survival in PCa.** (A) Nomogram constructed by integrating the BRPGs risk score with prostate-specific antigen value, Gleason score, and AJCC stage to estimate 1-year, 3-year, and 5-year biochemical recurrence-free survival probabilities in patients with PCa. (B) Calibration plots for 1-year, 3-year, and 5-year biochemical recurrence-free survival showing close agreement between nomogram-predicted and observed outcomes, indicating good calibration of the model. (C) Time-dependent receiver operating characteristic curves comparing the predictive performance of the nomogram with the clinically established models and individual clinicopathological variables at 1, 3, and 5 years. The nomogram showed superior discrimination across all evaluated time points, with area under the curve values of 0.789, 0.795, and 0.812 at 1, 3, and 5 years, respectively, exceeding those of the clinically established models and other individual predictors. **Abbreviations:** AJCC, American Joint Committee on Cancer; AUC, area under the curve; BCRFS, biochemical recurrence-free survival; BRPGs, Bregs-related prognostic genes; CEMs, clinically established models; PSA, prostate-specific antigen; ROC, receiver operating characteristic.

**Figure 6.**
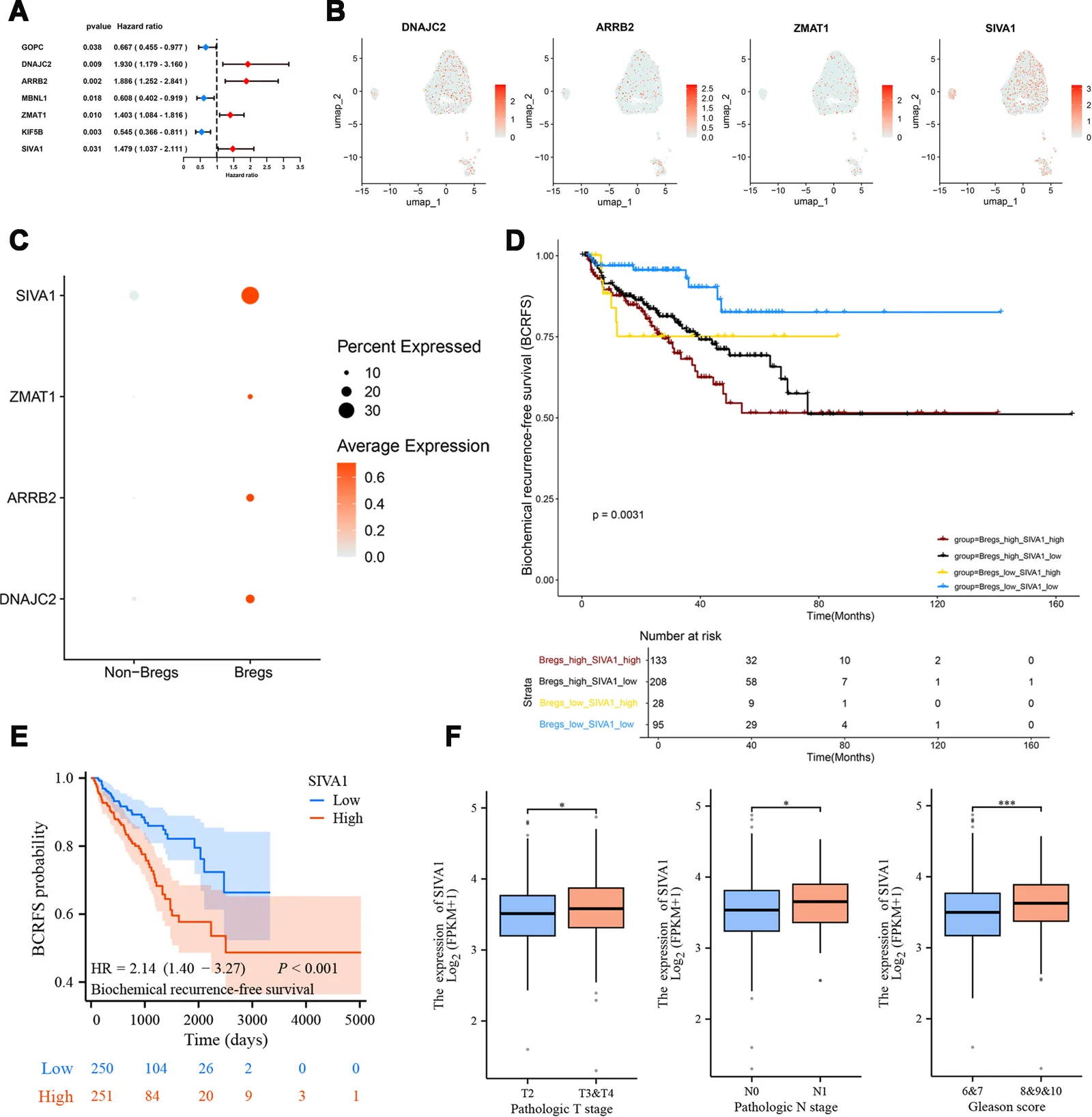
***SIVA1* emerges as a core BRPG gene associated with Bregs enrichment and unfavorable biochemical recurrence-free survival in PCa.** (A) Forest plot of univariate Cox regression analysis for selected genes included in the BRPG signature, showing hazard ratios with 95% confidence intervals. (B) UMAP feature plots showing the distribution of *DNAJC2, ARRB2, ZMAT1,* and *SIVA1* expression across B-cell subpopulations. (C) Dot plot comparing the expression of these four adverse prognostic genes between Bregs and non-Bregs; dot size represents the proportion of expressing cells, and color intensity indicates average expression. (D) Kaplan–Meier analysis of BCRFS stratified by combined Bregs score and *SIVA1* expression, showing the poorest outcome in the Bregs-high/*SIVA1*-high group and the most favorable outcome in the Bregs-low/*SIVA1*-low group. (E) Kaplan–Meier curve for BCRFS according to *SIVA1* expression, indicating significantly shorter BCRFS in patients with high *SIVA1* expression. (F) Box plots showing the association between *SIVA1* expression and clinicopathological characteristics, including pathological T stage, pathological N stage, and Gleason score, with higher *SIVA1* levels observed in more advanced disease categories. Statistical significance is indicated as *P* < 0.05 and **P < 0.001. **Abbreviations:** BCRFS, biochemical recurrence-free survival; Bregs, regulatory B cells; BRPGs, Bregs-related prognostic genes; FPKM, fragments per kilobase of transcript per million mapped reads; HR, hazard ratio; UMAP, Uniform Manifold Approximation and Projection.

### Development of a nomogram combining BRPGs and clinical features to enhance model utility

To enhance the clinical applicability of the risk model, we integrated BRPGs with clinical variables such as PSA levels, Gleason scores, and AJCC staging to construct a nomogram for predicting BCRFS in PCa patients (Figure 5A). Calibration curves were generated using the rms R package and bootstrap resampling. The results indicated that the calibration curves closely followed the 45-degree diagonal, demonstrating good agreement between the model’s predicted probabilities and the actual observed probabilities (Figure 5B). ROC analysis revealed that the area under the curve (AUC) values of the nomogram at 1, 3, and 5 years were 0.789, 0.795, and 0.812, respectively. The AUC values of the CEMs model at 1, 3, and 5 years were 0.776, 0.759, and 0.792, respectively. The nomogram outperformed the CEMs model at all time points and exhibited superior predictive accuracy compared to other clinical factors such as Gleason score, PSA, and stage (Figure 5C). The Brier scores of the nomogram at 1-year, 3-year, and 5-year follow-ups were 0.060, 0.147, and 0.198, respectively, indicating that the model possesses good predictive accuracy (Figure S3).

### SIVA1 as a core gene of BRPGs and its association with poor prognosis

To identify the key driving factors in the risk model, we conducted univariate Cox regression analyses on candidate genes of BRPGs, revealing that DNAJC2, ARRB2, ZMAT1, and SIVA1 were significantly associated with high-risk outcomes (Figure 6A). Additionally, both univariate and multivariate analyses were performed on all 20 functional hub genes (Figure S4). These supplementary analyses were included to provide interpretive support for the prognostic relevance of the functional hub genes and to assess whether selected genes maintained independent prognostic associations beyond the composite BRPG model. UMAP and bubble plots demonstrated that these four genes exhibited high expression levels in the Bregs subpopulation, with *SIVA1* showing significantly elevated expression compared to the other three genes (Figure 6B and C). Further evaluation utilizing single-cell RNA-seq data (Figure S5) confirmed that *SIVA1* is relatively enriched in stromal cell compartments. Stratification by Bregs score and *SIVA1* expression effectively distinguished patient BCRFS, with the Bregs high + SIVA1 high group exhibiting the shortest BCRFS and the Bregs low + *SIVA1* low group showing the longest (Figure 6D). Furthermore, the expression of *SIVA1* was positively correlated with the Bregs subpopulation (Figure S6), reinforcing its identification as a core gene. Kaplan-Meier analysis across multiple public datasets, including TCGA, consistently indicated that high *SIVA1* expression correlated with shorter BCRFS, although statistical significance was not attained in all cohorts (Figure 6E, Figure S7). Additionally, *SIVA1* expression was significantly correlated with pathological N staging, T staging, and Gleason scores, suggesting its involvement in the malignant progression of PCa and potential association with Bregs enrichment (Figure 6F). We observed significant upregulation of *SIVA1* expression in PCa cell lines, such as NCI-H660, further substantiating its potential role in aggressive PCa subtypes (Figure S8A-B). Notably, *SIVA1* expression was higher in the neuroendocrine PCa (NEPCa) subgroup compared to other subgroups, indicating its association with the aggressive characteristics of PCa. Furthermore, elevated expression levels were also observed in the Progenitor-like and KRT7 subgroups, suggesting that *SIVA1* may play a role in proliferating and progenitor cells (Figure S8C). In the HuPSA dataset, *SIVA1* was highly expressed in aggressive cell populations, including Progenitor-like, AdPCa AR+, and NEPCa, further supporting its potential role in these cell types (see Figure S8D-F). In the MoPSA dataset, we identified differential expression of *SIVA1* across several cell populations, underscoring its potential involvement in immune regulation and the cancer microenvironment, particularly within NEPCa and Proliferating subgroups (Figure S8A-H).

### SIVA1 expression, its association with LAG3, and poor prognosis in PCa

To further evaluate the clinical and biological relevance of SIVA1 in PCa, we analyzed SIVA1 expression using an IHC microarray that included Gleason scores and BCRFS-related follow-up data. In our TMA cohort, SIVA1 was broadly expressed in PCa tissues compared to adjacent normal tissues (Figure 7A, B). Although the difference did not reach statistical significance, patients with high SIVA1 expression exhibited a trend toward shorter **BCRFS** (Figure 7C), suggesting its potential role as a prognostic biomarker. Moreover, SIVA1 expression was significantly higher in tumors with Gleason scores greater than 7 (Figure 7D and E), further indicating its association with more aggressive tumor phenotypes. We subsequently applied CellChat to compare cell–cell communication between Bregs-high and Bregs-low groups. In the low group, Bregs primarily interacted with non-Bregs and within their own cluster. In contrast, Bregs in the high-expression group demonstrated enhanced self-interactions and markedly increased communication with Tregs and CD8^+^T cells (Figure 7F–H). Furthermore, SIVA1 expression was positively correlated with the immune checkpoint LAG3, suggesting a potential association with immunosuppressive features in PCa. To investigate whether this association also exists within T cells, we analyzed the relationship between SIVA1 and LAG3 expressions in T cell populations using single-cell RNA-seq data. Correlation analysis revealed a statistically significant but negligible association (*R* ═ 0.007), indicating a lack of meaningful correlation (Figure S9A). Additionally, co-expression analysis demonstrated that only a small proportion of T cells co-express both genes (Figure S9B). These results imply that SIVA1 and LAG3 are unlikely to be directly co-regulated within T cells. High SIVA1 expression was also associated with increased Bregs–Tregs/CD8^+^T cell communication patterns in CellChat analysis, suggesting a link between SIVA1-high tumors and a more immunosuppressive microenvironment; however, these findings do not establish a direct Bregs-specific mechanism (Figure 7I and J). Western blot analysis indicated that SIVA1 protein expression was markedly elevated in PCa cell lines, particularly in C4-2 and C4-2B, compared to the benign prostatic epithelial cell line BPH-1 (Figure 7K). Quantitative analysis further confirmed the significant upregulation of SIVA1 in these aggressive PCa cell lines (Figure 7L). To investigate the potential regulatory relationship between SIVA1 and LAG3, we performed siRNA-mediated knockdown of SIVA1 in C4-2 and C4-2B cells. The results showed that silencing SIVA1 led to a concomitant reduction in LAG3 protein expression, suggesting that SIVA1 may be associated with LAG3 expression in PCa cells, supporting a tumor cell-associated relationship (Figure 7M).

**Figure 7.**
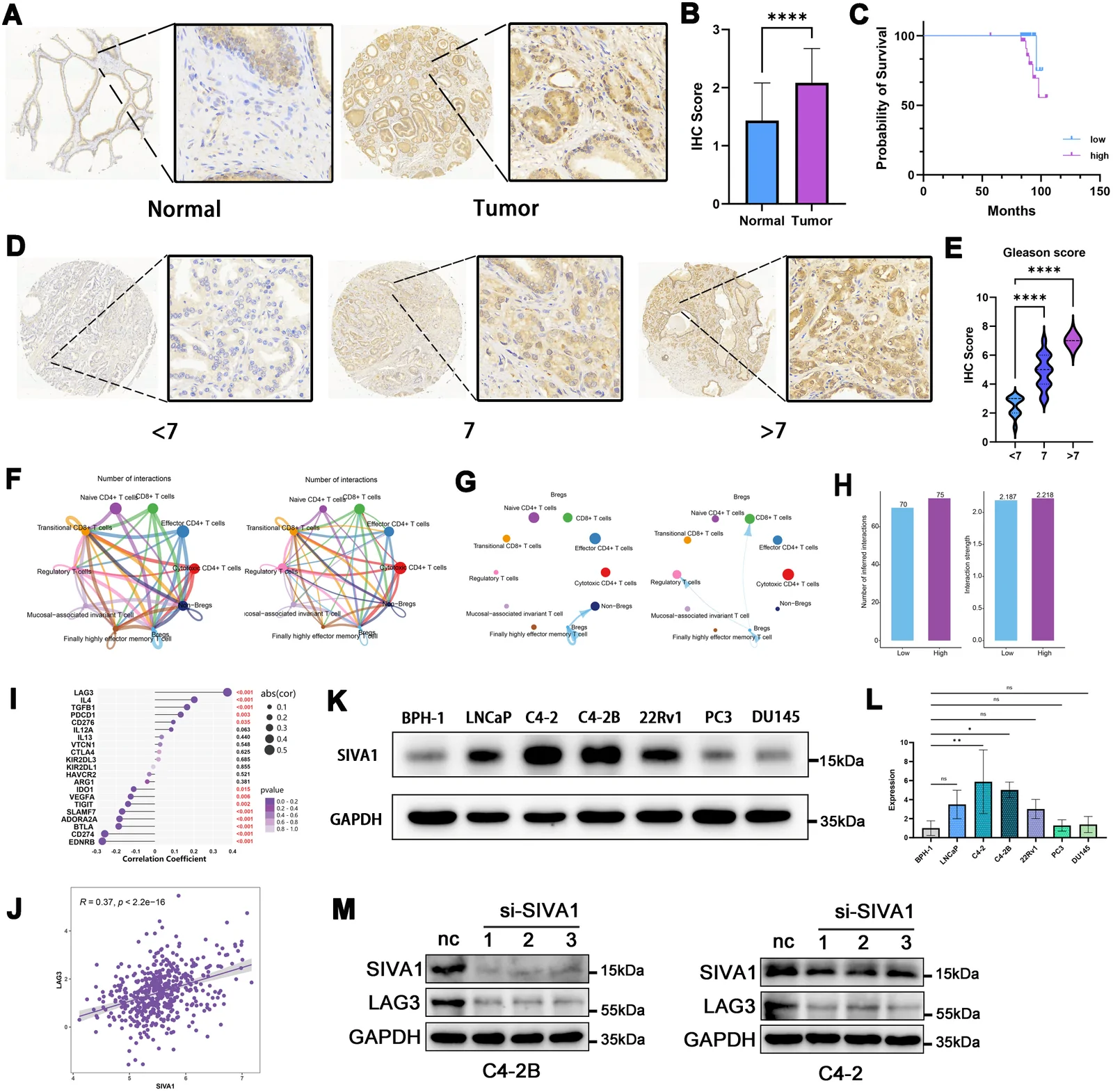
***SIVA1* is associated with LAG3 expression and immunosuppressive features in PCa.** (A) Representative IHC staining of *SIVA1* in adjacent normal prostate tissue and PCa tissue, with corresponding higher-magnification insets. (B) Quantification of IHC scores showing significantly higher *SIVA1* expression in tumor tissues than in paired normal tissues. (C) Kaplan–Meier analysis of BCRFS in the TMA cohort stratified by *SIVA1* expression, showing a trend toward shorter BCRFS in the high-*SIVA1* group. (D) Representative IHC staining of *SIVA1* in PCa tissues with Gleason scores <7, 7, and >7, with magnified insets showing progressively stronger staining in higher-grade tumors. (E) Violin plots of IHC scores across Gleason categories, demonstrating increased *SIVA1* expression in tumors with higher Gleason scores. (F) CellChat circle plots comparing the global communication networks between B-cell and T-cell subsets in the low- and high-Bregs groups. (G) CellChat plots centered on Bregs, showing that the high-Bregs group displays enhanced Bregs self-interactions and increased communication with regulatory T cells and CD8+ T-cell subsets. (H) Bar plots summarizing the total number and overall strength of inferred intercellular communications in the low- and high-Bregs groups. (I) Correlation analysis between SIVA1 and representative immune checkpoint genes, identifying LAG3 among the positively correlated inhibitory molecules. (J) Scatter plot showing a positive correlation between *SIVA1* and *LAG3* expression. (K) Western blot analysis of SIVA1 protein expression in the benign prostatic epithelial cell line BPH-1 and PCa cell lines LNCaP, C4-2, C4-2B, 22Rv1, PC3, and DU145, with GAPDH used as the loading control. (L) Densitometric quantification of SIVA1 protein levels across cell lines, confirming increased expression in PCa cell lines, particularly C4-2 and C4-2B, relative to BPH-1. (M) Western blot analysis of SIVA1 and LAG3 expression following siRNA-mediated knockdown of SIVA1 in C4-2B and C4-2 cells, showing concomitant reduction of LAG3 after SIVA1 silencing. Statistical significance is indicated as shown in the figure; bars represent mean ± SD where applicable. **Abbreviations:** BCRFS, biochemical recurrence-free survival; BPH-1, benign prostatic hyperplasia-1; Bregs, regulatory B cells; CD8, cluster of differentiation 8; GAPDH, glyceraldehyde-3-phosphate dehydrogenase; IHC, immunohistochemistry; LAG3, lymphocyte-activation gene 3; SD, standard deviation; siRNA, small interfering RNA; TMA, tissue microarray.

## Discussion

In this study, we identified a subpopulation of Bregs characterized by immunoregulatory properties and the enrichment of immunosuppressive pathways using single-cell transcriptomic data, pathway enrichment analysis, scoring methods, and the BayesPrism deconvolution algorithm. Utilizing the CellChat algorithm, we further analyzed the intercellular communication features between Bregs and other cell types within the PCa immune microenvironment. Importantly, we developed a nomogram for PCa and compared its performance with traditional CEMs. Our analysis of the AUC values for both the nomogram and the CEMs revealed that the nomogram outperformed the CEMs in predicting BCRFS in PCa. Additionally, we identified SIVA1 as a key gene within the Bregs subpopulation. Western blot and immunohistochemistry experiments further supported an association between SIVA1 expression and LAG3. Targeted intervention against SIVA1 in PCa cell lines resulted in reduced LAG3 expression, indicating a tumor cell-associated relationship between SIVA1 and LAG3 rather than a definitive Bregs-specific mechanism.

While the critical role of T cells in anti-tumor immunity is well-established in immune checkpoint blockade and adoptive T cell therapy, these advances also reveal the limitations of T cell responses, underscoring the need to explore complementary immune mechanisms [59]. Emerging evidence indicates that tumor-infiltrating B cells and plasma cells (PCs), collectively referred to as tumor-infiltrating B lymphocytes (TIL-B), play a significant and multifaceted role in the anti-tumor immune response [12, 40–42]. Similar to T cells, TIL-Bs exhibit diverse phenotypes, including effector B cells and Bregs. Bregs represent a unique immunosuppressive subset of B cells that exert immune regulation through interactions with specific immune cells and molecules in the TME, drawing increasing attention for their role in tumor immune escape. The predominant mechanism by which Bregs exert their effects is through the secretion of immunosuppressive cytokines, particularly IL-10, which enables Bregs to suppress immune responses across various cancers [20, 43, 44].

Studies have demonstrated that in lung cancer, ovarian adenocarcinoma, and gastric cancer, tumor cells release autophagosomes that induce B cell differentiation into IL10+ Bregs, inhibiting the immune responses of CD8+ and CD4+ T cells, thereby suppressing anti-tumor immunity [45]. In breast cancer, tumor cells prompt resting CD4+ T cells to differentiate into forkhead box P3 (FoxP3+) Tregs via tumor-induced regulatory B cells, promoting lung metastasis and highlighting the significant role of tumor-induced Bregs in immune escape and metastasis [46, 60]. Furthermore, IL-21-induced granzyme B (GrB+) Bregs exert immunosuppressive effects in the TME through GrB-dependent mechanisms, limiting T cell proliferation and inhibiting anti-tumor immune responses [27]. In line with these findings, our study provides experimental evidence to support the immunosuppressive potential of Bregs in PCa. Specifically, western blot analysis demonstrated that the knockdown of SIVA1 in PCa cells resulted in a concomitant reduction of LAG3 protein expression, suggesting a possible regulatory relationship between SIVA1 and the immune checkpoint molecule LAG3. This result aligns with our bioinformatic prediction linking SIVA1 to immunosuppressive features in PCa. However, the current data provide stronger evidence for a tumor cell-associated relationship between SIVA1 and LAG3, rather than a definitive Bregs-specific SIVA1–LAG3 mechanism. These findings further emphasize the relevance of Bregs in the PCa microenvironment while indicating that the SIVA1–LAG3 relationship observed here is best interpreted at the tumor cell level. These observations may inform future studies on Bregs-related immune regulation and SIVA1-targeted therapeutic strategies.

In this study, we identified a B cell subpopulation with immunoregulatory functions by analyzing scRNA-seq data from PCa tissues [20, 43, 44]. This subpopulation exhibited significantly elevated expression of the Breg-related surface molecule IL-10, consistent with previous descriptions of Bregs. Transcriptomic analysis revealed significant enrichment of immune-suppressive gene pathways associated with B cell function, suggesting its potential role in facilitating PCa progression. We further applied the BayesPrism algorithm for deconvolution of B cell subpopulations and analyzed TCGA PCa patient data to evaluate B cell infiltration levels. The findings indicated that Bregs comprised a higher proportion of B cell infiltration compared to other subpopulations. Additionally, the extent of Breg infiltration was strongly correlated with poor prognosis and various clinical features in patients. This observation aligns with prior reports indicating that the frequency of IL10+ B cells in PCa tissues is elevated and positively correlates with disease progression [33]. Notably, studies in mouse models suggest that immunosuppressive B cells promote PCa progression through the activation of the IKKα/STAT3 pathway [33, 61], and the elimination of immunoglobulin A (IgA)+IL10+programmed death-ligand 1 (PDL1)+ B cells significantly enhances CD8+ T cell anti-tumor activity, further substantiating the critical role of Bregs in tumor immune escape [20, 62].

To further investigate the molecular characteristics of the Breg subpopulation and construct an effective prognostic model, we employed the hdWGCNA analysis method to identify gene modules significantly associated with Bregs. By intersecting genes from significant modules with differentially expressed genes in Bregs, we identified key molecules for constructing the BRPGs risk model, which was validated in TCGA and other independent cohorts. The results demonstrated that this risk model effectively stratified patients into high- and low-risk groups, with the high-risk group exhibiting significantly poorer prognosis, underscoring its strong predictive value for PCa prognosis. During model construction, the SIVA1 gene emerged as a core gene. SIVA1 (Apoptosis-Inducing Factor) is a known pro-apoptotic regulator whose dual role in cancer and immune homeostasis has garnered considerable attention [63]. Early studies indicated that SIVA1 is a transcriptional target of p53 and promotes the ubiquitination and degradation of p53 through interaction with Hdm2, thereby inhibiting p53-mediated apoptosis [64]. Additionally, SIVA1 functions as an E3 ubiquitin ligase for p14ARF, facilitating its degradation and indirectly accelerating p53 inactivation [65], a mechanism that promotes cell proliferation and tumorigenesis. Furthermore, the role of SIVA1 in T cells has also been highlighted. Research indicates that SIVA1 negatively regulates the nuclear factor kappa B (NF-κB) signaling pathway and promotes T-cell receptor (TCR)-mediated T cell apoptosis. Upon deletion of SIVA1, NF-κB activity in T cells significantly increases, enhancing the expression of anti-apoptotic genes (e.g., Bcl-xL, c-FLIP), which may disrupt peripheral tolerance and affect immune tolerance [66]. Moreover, SIVA1 regulates microtubule dynamics to inhibit tumor cell migration and epithelial-mesenchymal transition (EMT), and its downregulation may promote tumor cell metastasis, emphasizing its critical role in inhibiting cancer spread [67]. In summary, SIVA1 modulates both the p53 pathway and EMT, limiting T cell apoptosis, thereby playing a crucial role in tumor cell proliferation and immune regulation in cancer progression. These findings provide a new theoretical basis for targeting SIVA1 as a potential therapeutic target for cancer treatment.

This study, for the first time, reveals that high expression of SIVA1 is closely associated with shorter BCRFS and an immunosuppressive profile in PCa patients. We also identified a significant positive correlation between SIVA1 expression and the immune checkpoint molecule LAG3. LAG3 (Lymphocyte-Activation Gene 3) is a key immune checkpoint molecule expressed on activated B cells, T cells, and NK cells, primarily regulating immune responses, particularly in tumor immune escape [68–70]. LAG3 inhibits T cell function by binding to its ligand Major Histocompatibility Complex II (MHCII), thereby suppressing T cell proliferation and effector function [71, 72]. This inhibitory effect is mediated through the binding of LAG3’s extracellular domains (such as D1 and D2) to the MHCII complex [73, 74]. Furthermore, LAG3 exacerbates immune suppression through interaction with tumor-associated ligands like fibrinogen-like protein 1 (FGL1) [75, 76]. In the TME, LAG3 promotes immune suppression in cooperation with Tregs, facilitating tumor immune escape [77, 78]. Clinically, LAG3 has been approved by the Food and Drug Administration (FDA) as an immune checkpoint receptor for treating advanced melanoma, demonstrating improved progression-free survival (PFS) and a favorable safety profile [68, 79, 80]. In this study, we observed extensive communication between Bregs, Tregs, and CD8+ T cells, as well as a significant positive correlation between the key Breg gene SIVA1 and LAG3. This suggests that SIVA1 and LAG3 are associated within a broader suppressive context. Their correlation, along with the CellChat results, is consistent with the possibility that SIVA1-high tumors exist within a more immunosuppressive microenvironment; however, these findings do not demonstrate that SIVA1 directly influences Bregs–Treg/CD8^+^ T cell interactions. Direct mechanistic evidence for a causal SIVA1–LAG3 axis remains to be established. Although the specific signaling relationship between SIVA1 and LAG3 requires further validation, their association implies that SIVA1 may contribute to the immunosuppressive characteristics of the PCa microenvironment. Currently, this role appears to be supported primarily at the tumor cell level, rather than as a direct mechanistic link within Bregs. Importantly, the siRNA and western blot experiments conducted in this study were performed in PCa cell lines rather than immune cells. Thus, our findings support a tumor cell-level association between SIVA1 and LAG3 but do not confirm a direct immune-cell mechanism. Further studies are needed to elucidate whether SIVA1 directly impacts immune checkpoint biology in PCa.

Based on these findings, both Breg subpopulations and SIVA1 may serve as novel therapeutic targets for PCa. Immunotherapeutic strategies targeting Bregs may include B cell depletion (e.g., utilizing a CD20 antibody) or inhibition of the immunosuppressive factors they produce (e.g., IL10, IL35). For instance, studies have shown that rituximab (an anti-CD20 antibody) reduces B cell infiltration in PCa tissues [35], indicating that B cell depletion could disrupt the suppressive network and enhance anti-tumor immunity. Furthermore, as a regulator of cell proliferation and apoptosis, targeting SIVA1 could restore p53 function and enhance T cell anti-tumor responses [64, 65]. Although specific small-molecule inhibitors targeting SIVA1 are currently unavailable, its dual mechanisms of action suggest that targeting or modulating SIVA1 may represent a promising direction for future immune combination therapy strategies, pending further mechanistic validation. Future studies could explore the use of SIVA1 inhibitors in combination with immune checkpoint inhibitors (e.g., LAG3 antibodies) to synergistically counteract tumor immune escape.

## Conclusion

This study provides, for the first time, a comprehensive exploration of the immunoregulatory characteristics and prognostic relevance of Bregs subpopulations in PCa through single-cell transcriptomic analysis. Through intercellular communication and gene expression profiling, we identified SIVA1 as a Breg-associated gene and found that its high expression correlated with LAG3 expression, supporting a tumor cell-associated relationship with immunosuppressive features in PCa. Further clinical data analysis revealed that increased Breg infiltration is closely linked to poor prognosis in PCa patients, and the BRPGs effectively predicts BCRFS in these individuals. Utilizing machine learning algorithms, we developed a BRPGs risk model and validated its robustness and predictive performance across multiple external datasets. Additionally, immunohistochemical analysis demonstrated widespread expression of SIVA1 in PCa tissues, with its expression correlating with key clinicopathological features, including Gleason score and BCRFS. These findings further support a tumor cell-associated link between SIVA1 and immunosuppressive features in PCa, while not establishing a definitive Breg-specific mechanism.

Moreover, the role of the SIVA1 gene extends beyond regulating cell proliferation and apoptosis; it may also influence T cell immune responses, suggesting that SIVA1 could represent a potential target for future immunotherapy in PCa, pending further mechanistic validation. Consequently, Breg-related features and SIVA1 may represent candidates for future investigation in PCa, particularly in immune cell contexts. These results provide new insights into the immune microenvironment of PCa and suggest potential directions for future immunotherapeutic research, highlighting the necessity for further experimental validation.

## Supplemental data

Supplemental data are available at the following link: https://www.bjbms.org/ojs/index.php/bjbms/article/view/14056/4169.

## Data Availability

All datasets analyzed in this study are publicly available. The TCGA-PRAD dataset was obtained from the GDC portal (https://portal.gdc.cancer.gov/). GEO datasets (GSE21034, GSE54460, GSE107299, GSE70769, and GSE94767) were downloaded from the NCBI GEO database (https://www.ncbi.nlm.nih.gov/geo/). The E-MTAB-6128 dataset was retrieved from ArrayExpress (https://www.ebi.ac.uk/arrayexpress/), and the DKFZ cohort was obtained from cBioPortal (https://www.cbioportal.org/). The single-cell RNA-seq dataset GSE181294 was obtained from the GEO database. The immunohistochemistry (IHC) data generated in this study are available from the corresponding author upon reasonable request. All dataset accession numbers and detailed information are provided in the Methods and References sections of the manuscript.
